# A Chick Model of Retinal Detachment: Cone Rich and Novel

**DOI:** 10.1371/journal.pone.0044257

**Published:** 2012-09-06

**Authors:** Colleen M. Cebulla, Chris P. Zelinka, Melissa A. Scott, Martin Lubow, Amanda Bingham, Stephen Rasiah, Ashraf M. Mahmoud, Andy J. Fischer

**Affiliations:** 1 Havener Eye Institute, Department of Ophthalmology and Visual Sciences, The Ohio State University, Columbus, Ohio, United States of America; 2 Department of Neuroscience, The Ohio State University, Columbus, Ohio, United States of America; University of Florida, United States of America

## Abstract

**Background:**

Development of retinal detachment models in small animals can be difficult and expensive. Here we create and characterize a novel, cone-rich retinal detachment (RD) model in the chick.

**Methodology/Principal Findings:**

Retinal detachments were created in chicks between postnatal days 7 and 21 by subretinal injections of either saline (SA) or hyaluronic acid (HA). Injections were performed through a dilated pupil with observation via surgical microscope, using the fellow eye as a control. Immunohistochemical analyses were performed at days 1, 3, 7, 10 and 14 after retinal detachment to evaluate the cellular responses of photoreceptors, Müller glia, microglia and nonastrocytic inner retinal glia (NIRG). Cell proliferation was detected with bromodeoxyuridine (BrdU)-incorporation and by the expression of proliferating cell nuclear antigen (PCNA). Cell death was detected with terminal deoxynucleotidyl transferase dUTP nick end labeling (TUNEL). As in mammalian models of RD, there is shortening of photoreceptor outer segments and mis-trafficking of photoreceptor opsins in areas of RD. Photoreceptor cell death was maximal 1 day after RD, but continued until 14 days after RD. Müller glia up-regulated glial fibriliary acidic protein (GFAP), proliferated, showed interkinetic nuclear migration, and migrated to the subretinal space in areas of detachment. Microglia became reactive; they up-regulated CD45, acquired amoeboid morphology, and migrated toward outer retina in areas of RD. Reactive NIRG cells accumulated in detached areas.

**Conclusions/Significance:**

Subretinal injections of SA or HA in the chick eye successfully produced retinal detachments and cellular responses similar to those seen in standard mammalian models. Given the relatively large eye size, and considering the low cost, the chick model of RD offers advantages for high-throughput studies.

## Introduction

Retinal detachment (RD) is a clinically important cause of visual loss; it is common and it is destructive to vision and to the eye itself. Poor visual acuity resulting from RD has been studied in humans and animal models for decades [1]. Such models have included intravitreal injections of dispase, for enzyme disruption of basement membranes [2], [3], [4], subretinal injection of saline to create a transient RD [5] or hyaluronic acid for a chronic RD [6], [7], or intravitreal injection of cells (e.g., fibroblasts, macrophages, retinal pigment epithelial cells) [1], [8], [9], [10]. Currently, the subretinal injection of hyaluronic acid is a prevalent RD model and has helped to explain the cascade of events following RD that can lead to permanent vision loss [6].

Changes to the photoreceptors, glia, and macrophages/microglia appear to be critical in the pathobiology of RD. Specifically, the photoreceptor outer segments (OS) degenerate and many of the photoreceptors apoptose, resulting in thinning of the outer nuclear layer (ONL) [11], [12]. This apoptosis is maximal 3 days following a retinal detachment in several mammalian models [13]. Subsequent to photoreceptor damage, Müller glia proliferate, hypertrophy, with up-regulation of intermediate filaments [13], [14], [15], [16], and migrate to the outer nuclear layer (ONL) [17], [18], [19], [20], contributing to the destructive scar formation which is the hallmark of proliferative vitreoretinopathy [6], [21]. Müller processes extend beyond the outer limiting membrane (OLM)and limit re-growth of photoreceptor outer segments after the retina is re-attached [18]. In addition, macrophages and microglia become reactive and accumulate in significant numbers in the retina and subretinal space, and contribute to retinal pathophysiology following RD [19], [22], [23], [24], [25], [26], [27].

A wide variety of mammalian species have been used to model retinal detachments and proliferative vitreoretinopathy, including rabbits, cats, mice, and primates [13]. But, other than primates, these species do not have a cone-rich retina needed to model humans. One animal that does possess similar cone density is the ground squirrel (*Spermophilus beecheyi*) and it has been used as a model for RD [28], [29]. Unfortunately, the ground squirrel model has significant disadvantages in poor availability and difficult handling. For these practical reasons the identification of better cone-rich animal models of RD is important for study of this retinal disorder. In addition, a model which permits our better understanding of the molecular biology of macroglial and microglial cells, and of their responses to retinal damage and to progenitor cells will add special value [30], [31], [32], [33], [34], [35], [36].

The chick has been used to study the development of the visual system [37], [38] and, more recently, for studying retinal damage and potential for regeneration [30], [31], [32], [33], [34], [35], [36]. It is a diurnal species with a sophisticated visual system emphasizing color vision. Chick retina contains four single cones responsible for color vision and one double cone, which may mediate achromatic motion perception [39]. The cone types include those that express visual pigments sensitive to long- (L), medium- (M), or short- (S) wavelengths. By convention, the chick L cone photopigment absorption peaks at 517 nm (also known as chicken red), M2 cone photopigment at 508 nm (chicken green), M1 cone photopigment at 455 nm (chicken blue), and S cone photopigment at 415 nm (chicken violet, the most similar to human S cones) [40]. Rods contain rhodopsin visual pigment (absorption around 500 nm) [40], [41]. Cones outnumber rods 6∶1 in the chick retina [42], while humans have approximately 20 times more rods than cones [43]. Cones are distributed throughout the entire chick retina with the highest density in the rod-free area centralis, which is analogous to the fovea centralis in humans [38], [41].

With this study we want to add further value to the use of the chick in biological research. We propose to develop a new chick model of RD using subretinal injection of hyaluronic acid. We hope to use its many modeling advantages which include (1) a cone-rich retina, (2) novel retinal glia [present in non human primates but not in rodents], (3) the wealth of data on micro and macroglial molecular responses to retinal damage and retinal regeneration, (4) the availability of mutant ocular strains, (5) a sequenced genome, (6) low cost, (7) widespread availability, (8) ease of handling, and (9) large eyes amenable to experimental manipulation.

## Results

**Figure 1:**
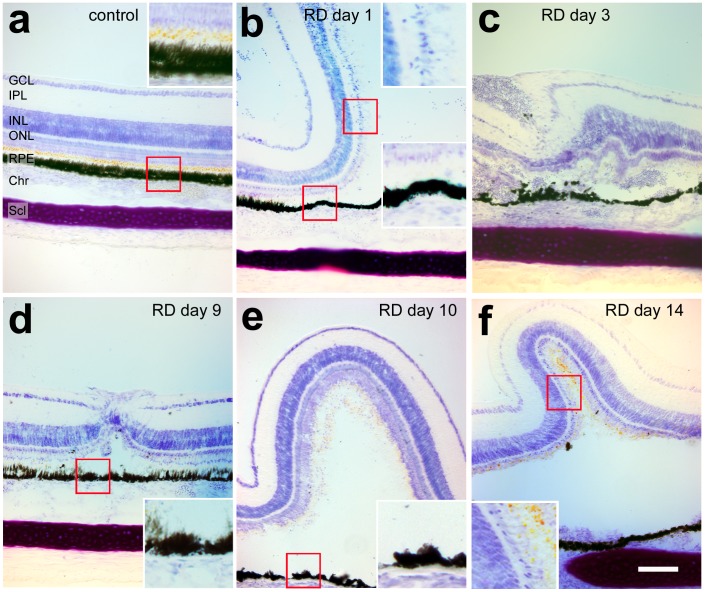
Altered outer retinal morphology in chick retinal detachment. Toluidine blue staining timecourse of retinal detachment frozen sections days 1, 3, 9, 10, and 14, showing the typical degenerative changes after HA RD as well as variability of the detachments generated. (a) Normal retina with details of outer retina, including oil droplets (predominantly orange-colored spheres) between the photoreceptor inner and outer segments and RPE apical processes (inset). (b–f) There is shortening of the outer segments and flattening of the RPE in areas of RD. Later there is a decrease in cell bodies in the outer nuclear layer (f). RPE hyperplasia is noted in some sections (C). ONL (outer nuclear layer), OPL (outer plexiform layer), PR (photoreceptor), IS (inner segments), OS (outer segments), RBC (red blood cells). Scale bar (50 microns).

Retinal detachments and controls were examined at days 1, 3–4, 7, 9–10, and 14–16. The RPE initially appeared flattened at day 1, with loss of apical processes (Figs. 1a–c). By 9 days after treatment, the detached RPE had a mounded appearance (Figs. 1d–f) with signs of hyperplasia in some samples (Fig. 1c). The photoreceptor outer segments were shorter and degenerated by day 1, compared to controls (Figure 1). In addition, we observed thinning of the outer nuclear layer (ONL), suggesting loss of photoreceptors (Fig. 1f). Saline detachments had resolved by the day 3 timepoint (not shown), while the hyaluronic acid detachments persisted.

**Figure 2:**
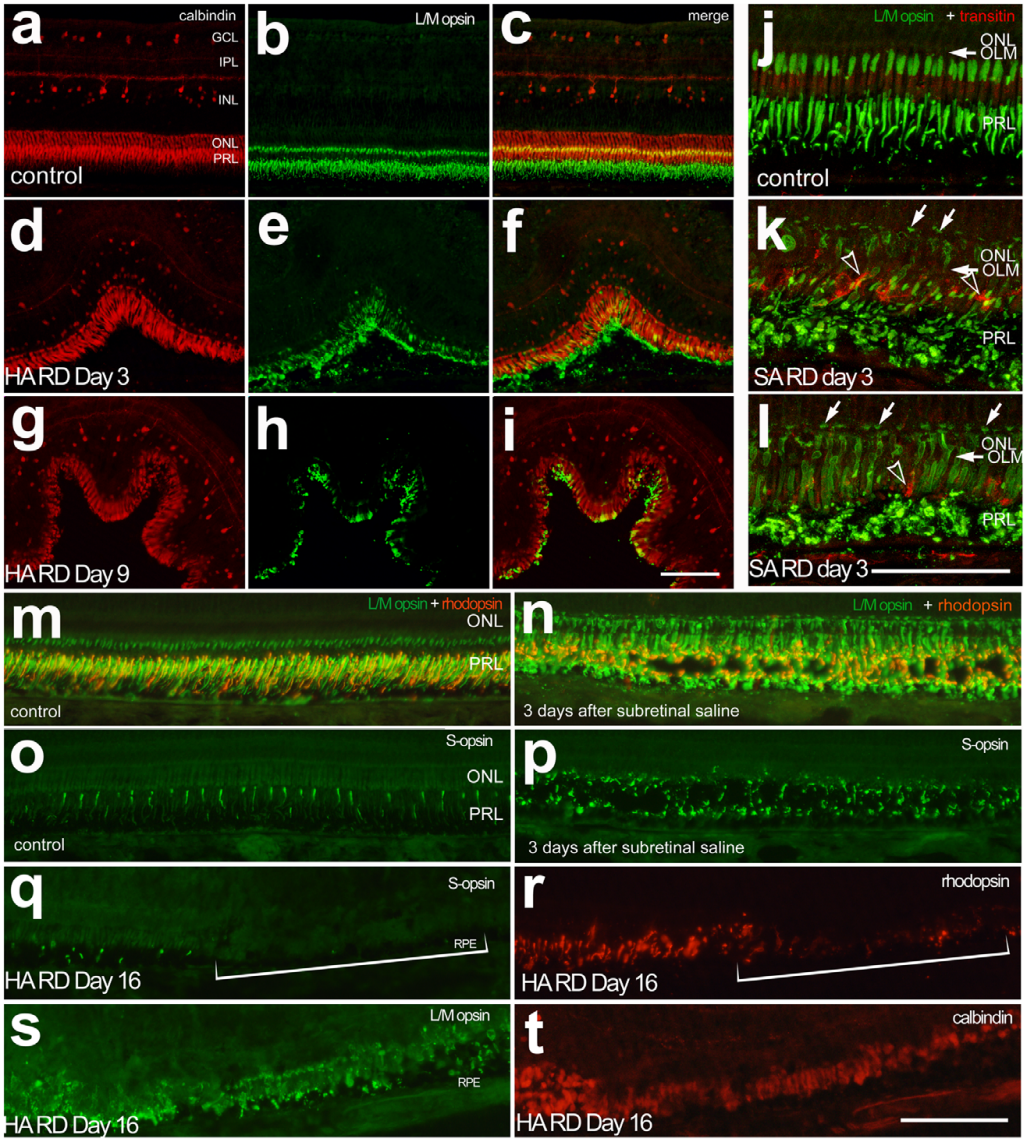
Mistrafficking of photoreceptor opsins in RD. Calbindin (red) and L/M opsin (green) immunostaining in control retina of the untreated fellow eye (a–c) and hyaluronic acid RD at day 3 (d–f) and 9 (g–i). Note decreased intensity of L/M opsin staining at day 9 HA RDs. Confocal microscopy demonstrates early mistrafficking of L/M opsin (green) with staining of the photoreceptor cell body and cone pedicles (arrows, k, l) in SA RDs at day 3 compared to untreated controls. The L/M opsin staining is present in areas consistent with the cone IS/OS as well as the cone ellipsoids in normal chick retina (j). Some transitin-positive glial processes are seen extending beneath the outer limiting membrane (OLM, arrowhead k, l). Similar to day 3 HA RDs, day 3 SA RDs show mistrafficking of rhodopsin (red) and L/M opsin (green, m, n) and S opsin (green, o, p), despite resolution of subretinal fluid. In advanced HA RD (Day 16 RD, q–t) there is redistribution of L/M opsin and calbindin to the cell body with loss of S opsin and rhodopsin (bracket). ONL (outer nuclear layer), OPL (outer plexiform layer), PRL (photoreceptor layer). Scale bar 50 microns.

Immunohistochemical analyses of photoreceptors showed mis-trafficking and disorganization of L/M opsin, S opsin, and rhodopsin in areas of retinal detachment (Figure 2). These changes were similar in both saline (SA) and hyaluronic acid (HA) RDs even though SA RDs had resolved by day 3. Re-distribution of L/M opsin to the photoreceptor cell body was seen using confocal microscopy. Opsin-immunofluorescence was present across the inner segments, cell bodies and axon terminals of stressed photoreceptors in regions of detachment (Figs. 2j–l). At approximately RD day 9, there was reduction in rhodopsin and S opsin levels, with relative preservation of L/M opsin and calbindin (Fig. 2). Labeling for S opsin and rhodopsin were reduced in detached regions of retina, whereas labeling for L/M opsin and calbindin remained, but appeared disorganized (Figure 2q–t).

**Figure 3:**
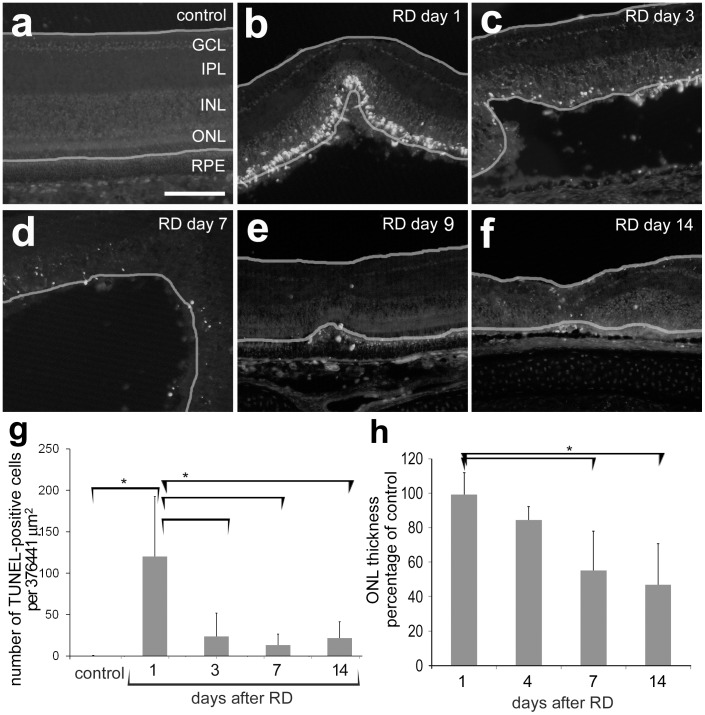
Photoreceptor cell death in chick RD. (a–f) Representative photographs of TUNEL-positive cells in control retina and day 1, 3, 7, 9 and 14 HA RDs. TUNEL positive cells are more prevalent in the outer nuclear layer early after detachment. The yellow lines mark the inner and outer limiting membranes. (g) Quantification of TUNEL: a bracket with an asterisk marks statistically significant differences between control and day 1 (p<0.0001) as well as day 1 and day 3 (p<0.0001), day 7 (p<0.0001), and day 14 (p = 0.0004). (h) Quantification of outer nuclear layer thickness: a bracket with an asterisk marks statistically significant differences between day 1 and day 7 (p = 0.0005) and day 14 (p = 0.0033). Error bars represent standard deviation.

To evaluate the timecourse of photoreceptor death in this model, TUNEL analysis was performed. Cell death was significantly increased in detached retina (ANOVA, p = <0.0001, Figure 3). Cell death was maximal at day 1 after detachment and then decreased over time (day 1 control: day 1 RD (p<0.0001), day 1 RD: day 3 RD (p<0.0001), day 1 RD: day 7 RD (p<0.0001) and day1 RD: day 14 RD (p = 0.0004)). TUNEL labeling was not significantly different than in controls at day 3, 7, and 14. The labeling appears primarily in the outer nuclear layer (ONL) but some cells were seen in the INL (data not shown). ONL thickness, a measure of photoreceptor cell survival, was unchanged at day one, but decreased significantly at later timepoints following retinal detachment, measuring 99% of paired control at detachment day 1, 84% at day 3, 55% at day 7, and 47% at 14 days following retinal detachment (Figure 3, ANOVA; p = 0.0002 ). The mean ONL thickness in regions of detachment was significantly less than in paired controls at day 14 (p<0.0001) and borderline at day 7 (p = 0.0088). ONL thickness in day 1 RDs was significantly greater compared to days 7 and 14 (p = 0.0005 and 0.0033, respectively).

**Figure 4:**
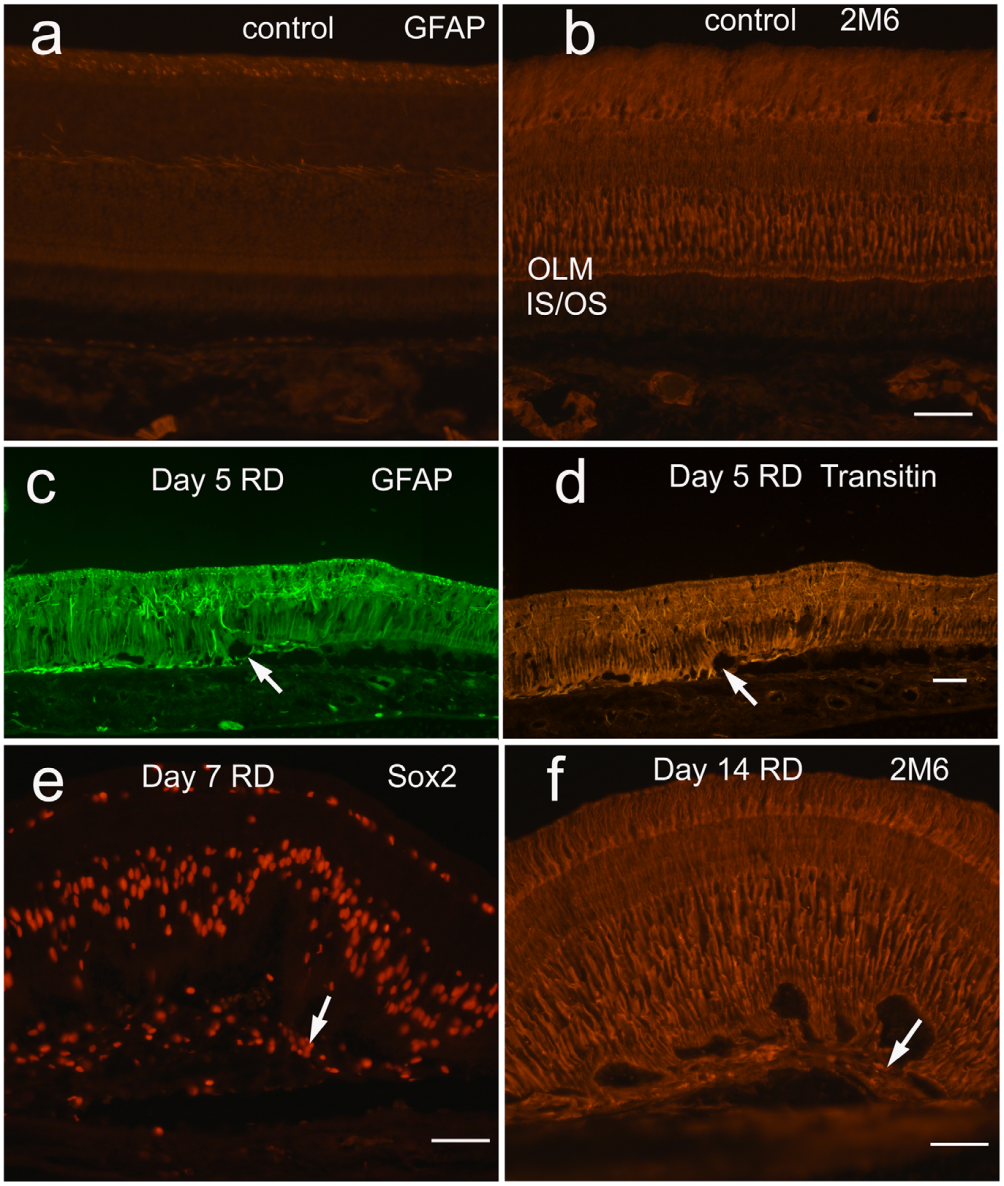
Müller glia activated in chick RD: Expression of intermediate filaments GFAP (untreated (a), day 5 HA RD (c)) and transitin, the chick homologue of nestin, (day 5 HA RD (d), untreated not shown) are essentially negative in normal control retina and increase after RD by day 3 (not shown). GFAP- and transitin-positive Müller glial processes extend beneath the outer limiting membrane (OLM) in HA RDs (arrows c, d) to the subretinal space. The Müller marker TOP_AP_ (antibody 2M6) is present primarily in a vertical distribution throughout the untreated retina in Müller glia (b) and increases particularly in the outer retina below the OLM in HA RDs, with accompanying undulation of the outer retina (f). Cells in subretinal scars are positive for Müller progenitor marker Sox2 (arrow, e) and 2M6 (arrow, f).

The cellular responses of the Müller glia to RD were evaluated. Similar to findings in mammalian models, the expression of the intermediate filament GFAP increased in regions of detachment (Figure 4) as early as 3 days after detachment. Expression of transitin, the avian homologue of mammalian nestin, was similarly increased (Fig. 4d). Glial processes positive for GFAP, transitin, and Müller glial marker TOP_AP_ (topographic marker expressed along the anterior-posterior axis, detected by antibody 2M6) [44] were seen extending beyond the outer limiting membrane (OLM) in areas of RD as early as day 3 (Figs. 2, 4). Areas consistent with subretinal scar were visualized at day 7 and 14 with large clusters of subretinal glial processes, as well as cell bodies positive for Sox2 and TOP_AP_ (Fig. 4).

**Figure 5:**
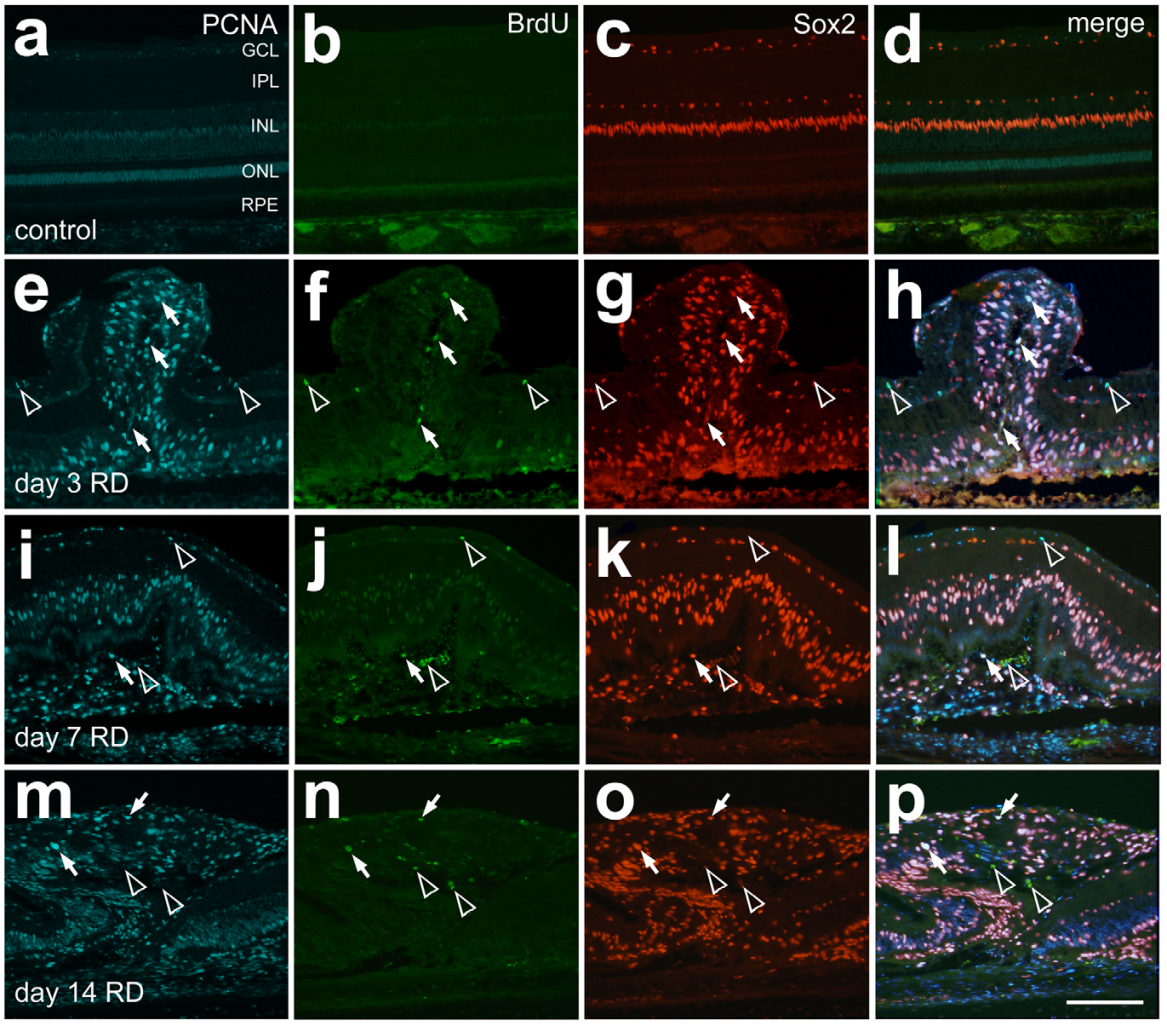
Müller glia proliferate after HA RD. Immunostaining to detect proliferating cells demonstrated more PCNA (blue) than BrdU (green) positive cells at the timepoints tested after RD (day 3, 7, 14). Sox2 (red) labels nuclei of Müller glia and NIRG cells. Merged images of PCNA, Sox2, and BrdU demonstrate a good correlation of PCNA and Sox2, including in subretinal scar. Cells labeling with all three markers are marked with arrows, while Sox2 negative cells labeling with PCNA and BrdU are marked with open arrowheads.

Cell proliferation, particularly of Müller glia, has been reported following retinal detachment in mammalian models [13], [45], [46]. Figure 5 shows proliferating cells present in detached retina; most were Sox2/PCNA-positive Müller glia (Fig. 5). PCNA-positive cells were more numerous than BrdU-positive cells and predominantly co-localized with Sox2, but not the NIRG marker homeobox proteinNkx-2.2 (Nkx2.2), thus indicating Müller glia proliferation (Fig. 5). Müller glia also exhibited interkinetic nuclear migration in regions of detachment, with migration of nuclei into the outer retina (Figs. 4–5).

**Figure 6:**
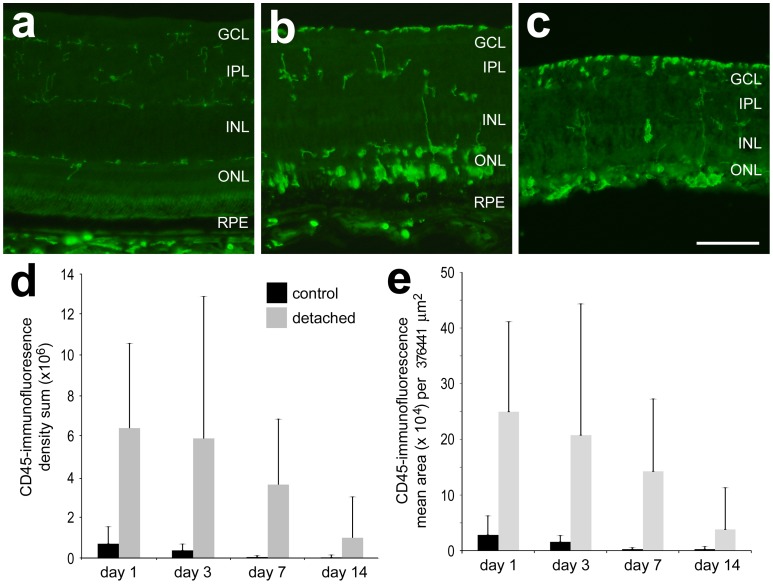
Microglia are up-regulated in chick SA and HA RD. SA RDs (b) and HA RDs (c) show up-regulation of microglial marker CD45 (green) in areas of RD compared to control retina (a). The SA RD is resolved by day 3. The microglia acquire amoeboid morphology and migrate to the outer retina/subretinal space. Quantitation of CD45 staining in HA RD over time (d, density sum and e, mean area).

A highly localized infiltration and activation of retinal and subretinal microglia and macrophages is characteristic in mammalian models of RD [22], [24], [25]. In the chick RD eyes, there was a significant accumulation of CD45-positive microglia/macrophages in the detached retina compared to controls (Fig. 6). Quantification of CD45-immunofluorescence indicated significantly increased mean area of signal per field of view from 2.8×10^4^ relative intensity units in control retina to 25.0×10^4^ in RD retina at day 1 (p = 0.0092), and the CD45 density sum increased from 7.2×10^5^ to 64.2×10^5^ (p = 0.0165). Density sum was significantly elevated in day 3 RD retina at 59.0×10^5^ compared to day 3 controls at 4.0×10^5^ (p = 0.0118), the other days did not reach significance after Bonferroni correction.

**Figure 7:**
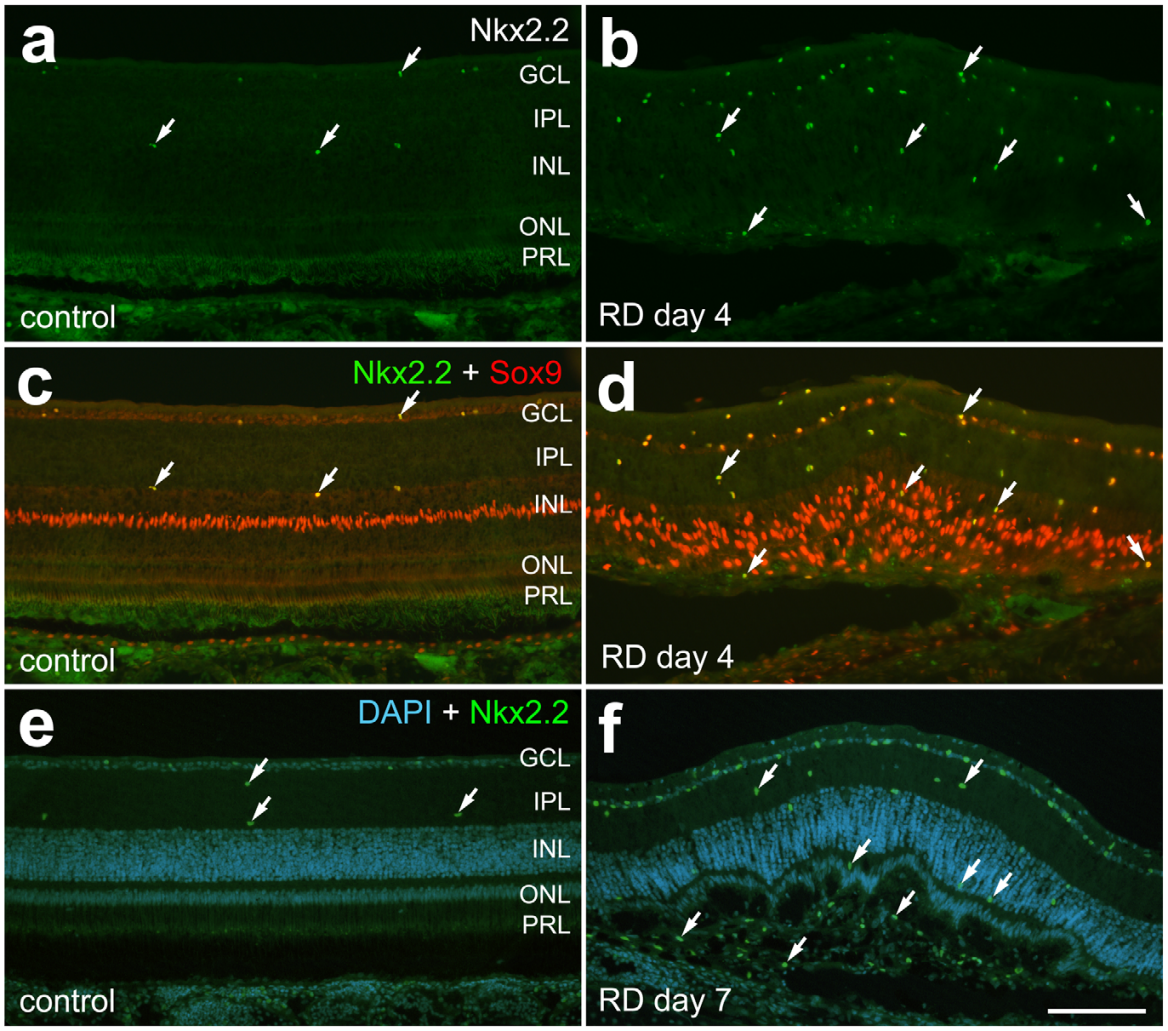
NIRG cells are mildly up-regulated after chick HA RD. NIRG cells (arrows) are positive for Nkx2.2 (green, a–f) and Sox9 (orange, c, d) and are increased after HA RD compared to controls. The up-regulation of NIRGs is less than that of Müller glia (Sox9+/Nkx- cells in the inner nuclear layer and outer retina). NIRGs were identified in subretinal scars (f, arrow; green = Nkx, blue = DAPI).

Recently, a novel population of glial cells (nonastrocytic inner retinal glia (NIRG) has been detected in the retinas of multiple species, including chick and primates [34], [47], and seen particularly after retinal damage in regions of focal detachment [34]. NIRG cells are known to express Sox2, Sox9, Nkx2.2, transitin, and vimentin, but are negative for GFAP and Pax2 [34]. Immunostaining for NIRG cells revealed an accumulation of NIRG cells above background controls in areas of RD (Figure 7). Surprisingly, NIRG cells were detected in subretinal scars as well as inner retina.

## Discussion

To the best of our knowledge, this is the first study utilizing the chick as a model of retinal detachment. There are many biologic, logistic, and practical advantages of the cone rich chick model system. Briefly, they offer advantages in cones, glia, characterized retinal responses, sequenced genome, availability, low cost, ease of handling, and large eyes. While chicks are not generally selected based on genetic strain, there are some ophthalmic mutants available [as reviewed in [48]]. Since the eye is large, intravitreal injections can be performed easily for potential high-throughput analyses of therapeutic agents.

As an alternative to the ground squirrel, the chick compares favorably in terms of availability, handling, and cost. The chick and ground squirrel cones have similarities, but some differences (Table 1). Chick retina serves tetrachromatic vision and contains four single cones (L, M1, M2, and S sensitive) and one double cone [39]. Ground squirrels have dichromatic vision with cones most sensitive to M (518 nm) and S (436 nm) wavelengths, as determined by ERG testing [49]. Similar to chicks, ground squirrels too are diurnal, with a very high cone density: about 7.5 million cones per retina. This is 1.6 times the human cone density (4.6 million cones) [50]. They have a retina in which 85% of the photoreceptors are cones, about 5.7 cones-per-rod [29]. In comparison, the chicks have 6 cones-per-rod [42]. The highest cone density in ground squirrel (49,550/mm^2^) is in the visual streak, analogous to the fovea centralis, in a horizontal strip of retina 2mm ventral to the optic nerve head [51]. In comparison, the chick area centralis is reported to have between 10,000–16,000 cones/mm2, although average cone density is reported higher by other studies [52]. Unlike mammalian cones, chick cones contain an oil droplet that filters out light below wavelengths 575, 520, 497, or 454 nm [39], [53]. Chick rods do not contain an oil droplet.

Despite some differences in their cone structure as compared with mammals, chick cones showed similar responses to retinal detachment. The photoreceptor outer segments demonstrated abnormalities in the detached areas as soon as one day after detachment. There was mis-trafficking of photoreceptor opsins and rhodopsin, similar to that in other models of detachment [28], [54], [55], [56], [57]. Cone opsins were redistributed to the plasma membrane as the outer segments degenerated. Similar to Sethi et al. [16], we found L/M opsin redistributing to the cell body in retinas with more severe degeneration in HA RDs. We also observed L/M opsin staining on the photoreceptor cell body and cone pedicles in the re-attached day 3 SA RD eyes.

The ground squirrel retina has been evaluated with histologic, molecular, and electrophysiologic techniques to determine rod and cone responses to RD. As in other mammalian species, outer segment degeneration began at 1 day. Rex et al. [56], [57] demonstrated a dramatic loss of mRNA and protein expression of cone opsins early after RD (days 1–3), with a relative preservation of rod opsin expression. Our studies indicate a longer retention of cone markers in the chick RD model with some L/M opsin and calbindin staining detectable in day 14 RDs. However, we note that S opsin-positive cones showed extreme down-regulation of these S photopigments in areas of severe degeneration, suggesting that S cones could be more sensitive to damage from RD. Nork et al. [58] demonstrated significant damage to S cones in human RDs. Experimental RD studies have yielded mixed results regarding whether S cones are more sensitive to RD damage. Future studies of S cone mapping should be done with the chick RD model to answer this question of relative stress sensitivity.

Although there is permanent loss of some photoreceptors following RD, re-attachment studies have demonstrated recovery of rods and cones, including re-expression of cone opsin [56], [57]. Sakai demonstrated similar transient depletion of S and M cone opsins in ground squirrel retinas that had been detached for 1 day and then reattached [59]. It is clinically and experimentally important that the time-course of cell death of photoreceptors following RD was similar in the chick model to that seen in human retinas. Dying cells were most abundant at day 1. This is earlier than the day 3 peak in cell death observed in several mammalian RD models [6]. The abundance of dying cells was highly variable between individuals, likely because the size of the detachment was variable. However, outer nuclear layer thickness, reflecting photoreceptor cell death, was initially preserved and did not decline significantly until days 7–14 after detachment. These data fit well with human data suggesting that the interval 7–30 days post-detachment is likely to be most significant for visual loss due to photoreceptor damage [60], [61], [62], [63], [64], [65]. The mechanism of visual loss in patients with macula-affecting RD is highly correlated with both photoreceptor cell death (indicated by thinning of the outer nuclear layer) and by abnormalities in the photoreceptor outer segments (indicated by disruption of the “intermediate line”) on spectral domain optical coherence tomography (SD-OCT) [66]. This disruption can improve in patients over time and fits with improving visual acuity [66]. Future studies in the chick, including use of SD-OCT, will be useful to better evaluate photoreceptor recovery after retinal detachment repair. Here, most experiments used subretinal hyaluronic acid which created a chronic detachment. In saline RDs, the detachments had resolved by day 3. However, in these re-attached saline RDs, responses were similar to those of hyaluronic acid RDs, including mistrafficking of photoreceptor proteins (Fig. 2k–l, m–p) and microglial activation and migration towards the subretinal space (Fig. 6b).

There is a local response of Müller glia in most mammalian models of RD and also in human retinectomy specimens [6], [16]. In contrast, the Müller glia in the ground squirrel are non-reactive in areas of RD [28]. This is an important research liability. The chick RD model is a better match for feline and human RD than is the ground squirrel in its prominent Müller glial activation, involving GFAP up-regulation, distal migration and proliferation. Similar to these other models, Müller processes extend beyond the OLM and appear to establish subretinal scars in some eyes. Recently, the Müller glia have been shown to proliferate, extend processes, and migrate beneath the retina to form subretinal scars [21]. Similarly, in the chick model we observed proliferation of Müller glia and interkinetic nuclear migration, with movement toward the outer retina, and extension of processes beyond the OLM. Histological examination of the subretinal scar in the chick indicates the accumulation of Müller glial processes and cell bodies. These results suggest that some Müller progenitor cells detach and migrate wholly into the subretinal space, while some only grow processes into the subretinal space. Such migration patterns and mechanisms are of special importance for translational research. Controlling Müller glial activation and gliosis can be critical to stopping subretinal scars and proliferative vitreoretinopathy.

Chick retina contains a recently-identified special glial cell type termed non-astrocytic inner retinal glia (NIRG). NIRG-like cells have been identified in the retinas of multiple species including dogs and monkeys, but not in the retinas of rodents [34], [47]. NIRGs activate and proliferate in response to chemical retinal injury with NMDA and IGF-1 in the chick, which leads to multiple small detachments of the retina [34]. In these detached areas Müller glia are absent, but an accumulation of NIRG cells fringe the Müller glia around damaged areas in the inner retina, vitread to where the Müller glia typically reside [34]. Little is known about the function of NIRG cells in response to retinal damage. To determine whether NIRG cells were prominent in the model of RD created here, we evaluated NIRG populations in the detached areas. These studies demonstrated a small increase in numbers of NIRG cells in the inner retina. However, Nkx2.2-negative Müller glia, expressing Sox2 and Sox9, were the predominant glial cell type in detached retina in this model. Surprisingly, some NIRG cells were detected in subretinal scars, along with Müller-glia derived cells (negative for Nkx and positive for Sox-9 as well as Sox-2, and TOP_AP_). These studies support the notion that macroglial cells contribute to subretinal scar formation after retinal detachment [21].

**Table 1:** Short-wavelength (S), Medium-wavelength (M), Long-wavelength (L), cones per square millimeter (c/mm^2^).

	Chick	Ground Squirrel	Human
Cone Vision	Tetrachromatic-S-M2-M1-L	Dichromatic-S-M	Trichromatic-S-M-L
Oil Droplet	Yes	No	No
High Acuity Vision	Area Centralis	Visual Streak	Fovea Centralis
Fovea	No	No	Yes
Average Retinal Cone density	10,000–35,961c/mm^2^ (a)	24,310–49,550c/mm^2^ (b)	2500c/mm^2^ peripheral 6000c/mm^2^ central (c)

a. Reviewed in [52]. Cone density varied widely between experiments.

b. Peak cone density was highest in the visual streak and lowest density in the dorso-nasal periphery [51].

c. Determined by histology from donor eyes [72].

Similar to other studies of mammalian models, the macrophage/microglial response to RD in the chick is dramatic. Microglia are resident tissue macrophages of the CNS. In the chick eye they are identified by immunostaining with CD45 [67]. In untreated eyes quiescent microglia are typically located in the plexiform layers of the retina. After RD, microglia migrate to the outer retina, up-regulate levels of CD45, acquire amoeboid morphology and enter the subretinal space. Nakazawa et al. [22] identified the importance of the macrophage product MCP-1 in decreasing photoreceptor survival in a murine model of RD. While the chick does not have a known MCP-1 homologue, it will be valuable to determine whether other specific macrophage products can reduce photoreceptor survival similar to those in mammalian models [22], and whether there is a role for microglia contributing to Müller glial proliferation and subretinal scar formation.

In conclusion, the chick model of RD shares many similarities with human, and advantages over other mammalian models. It is a cone-rich and research friendly model, and it could offer added insights into the pathobiology of retinal detachment. It could be helpful for high-throughput studies of neuroprotective or anti-proliferative agents that would ameliorate RD.

## Materials and Methods

### Animals

Leghorn chicks (*Gallus gallus domesticus*) were obtained from the Department of Animal Sciences at The Ohio State University. They were used at postnatal days 7 to 21 (P7–P21) to create the retinal detachment model under an IACUC approved protocol (#2009A0139). Only one eye was treated. The chicks were housed with free access to Purina chick starter and water in a 12 h light-to-dark cycle in a stainless steel brooder.

### Retinal Detachment Surgery

This research adheres to the principles of the ARVO Statement for the Use of Animals in Ophthalmic and Vision Research. Chicks (P7–21) were anesthetized with inhaled isofluorane via nonrebreather mask. Pupil dilation was achieved with multiple rounds of drops of tubocurarine hydrochloride (Sigma: 6.7 mg/ml in saline with 0.1% benzalkonium). Tubocurarine, a neuromuscular blocking agent that is a competitive antagonist of nicotinic neuromuscular acetylcholine receptors [68], was used for dilation since chick iris contains prevalent nicotinic cholinergic receptors [69].

Left eyes were prepped with betadine swabs and a pediatric lid speculum was inserted. A lateral canthotomy was created with a sterile razor blade for better exposure of the globe. The conjunctiva was stabilized with 0.12 forceps and then was dissected temporally with a 25 g needle tip to expose the sclera. A 25 g needle on a 5 cc syringe was inserted approximately 3 mm posterior to the limbus and vitreous was aspirated from the central vitreous cavity until the eye softened slightly. The subretinal injection was then performed through this opening with a custom blunt-tip 30 g needle on a Hamilton syringe (Hamilton Company, Reno, NV) by gently embedding the tip of the needle into the central retina. The tip was visualized through the pupil by using an operating microscope while the cornea was covered by a glass coverslip and Genteal gel. Subretinal injections of approximately 25 microliters of sterile saline (n = 3) or undiluted hyaluronic acid (Healon, 10 mg/ml; AMO, Santa Ana, CA; n = 32) were delivered to the left eye to bleb-up the retina. The eye was dressed with erythromycin ophthalmic ointment at the end of the procedure. Eyes were enucleated for analysis at day 1 (n = 7), 3–4 (n = 10), 5–7 (n = 6), 9–10 (n = 3), and 14–16 (n = 6).

### Enucleation and Histology

Animals were euthanized by CO_2_ gas inhalation followed by pneumothorax induction. Enucleation was performed by dissecting off the periocular tissue and muscle attachments to the globe with forceps and Wescott scissors and severing the optic nerve. The globe was processed, fixed and sectioned as previously described [70]. A razor blade was used to remove the anterior cap. The eye was placed in fixative containing phosphate buffer with 4% paraformaldehyde and 3% sucrose in 0.1 M phosphate buffer at pH 7.4 for 30 minutes. Eyes were washed twice in PBS for 10 minutes and placed in 30% sucrose in PBS overnight. The eyes were embedded in OCT (Optimal Cutting Temperature, Electron Microscopy Sciences) solution and snap frozen. Sections were consistently cut at 12 microns in the area of the RD for histologic analysis.

### BrdU Labeling

Intravitreal injections of 2 micrograms 5-bromo-20-deoxyurdine (BrdU; 100 microgram/mL; Sigma-Aldrich) were delivered approximately 4 hours prior to euthanization (n = 4 chicks at each timepoint: day 1, 3, 7, 14) as previously described [36]. Frozen sections were immunostained using the anti-BrdU antibody (G3G4, Developmental Studies Hybridoma Bank). BrdU positive cells were determined at different levels in the area of the retinal detachment to determine the number of proliferating cells.

### TUNEL Assay

Apoptosis was evaluated with the TUNEL assay with the In Situ Cell Death Kit (TMR red; 1215679910, Roche Applied Science) per the manufacturer’s instructions.

### Immunohistochemistry

Immunohistochemistry was performed to detect markers as previously described [34]. Antibodies to rhodopsin (Rho 4D2, mouse, generous gift Dr. R. Molday, University British Columbia), L/M opsin (AB5405, rabbit, Chemicon), S opsin (rabbit polyclonal anti-blue opsin, Millipore) were used to evaluate photoreceptor opsins. Mouse anti-calbindin (300; Swant Immunochemicals, Bellinzona, Switzerland) was used to detect photoreceptor calbindin. Antibodies to BrdU (G3G4, mouse, Developmental Studies Hybridoma Bank) and PCNA (M0879, mouse, DAKO) were used as markers of proliferating cells. CD45 (HIS-C7, mouse, Cedi Diagnostic) was a marker for microglia/macrophages [67], GFAP (Z0334, rabbit, Dako) and vimentin (H5, mouse, DSHB) were markers for Müller glia. Sox-2 (Y-17, goat, Santa Cruz), Sox-9 (AB5535, rabbit, Chemicon), and transitin (EAP3, mouse, Developmental Studies Hybridoma Bank) were used to identify NIRG cells and Müller glia. DRAQ-5 (Biostauts Limited) was used as a counter stain for nuclei for determination of outer nuclear layer thickness.

### Image Analysis

Photomicrographs were obtained using a Leica DM5000B fluorescent microscope and Leica DC500 digital camera. Confocal images were obtained using a Zeiss LSM 510 at the Hunt-Curtis Imaging Facility. Image analysis was previously described [34]. Images were optimized for color, brightness, and contrast, and double-labeled images overlaid by using Adobe PhotoshopTM 6.0. Cell counts were made from at least three different animals at the area of maximal detachment, and means and standard deviations calculated on data sets. To minimize the variability of region-specific differences within the retina, cell counts were made from the same region of retina for control and retinal detachment areas. Immunofluorescence was quantified by using Image-Pro 6.2. Identical illumination, microscope, and camera settings were used to obtain images for quantification. Fixed areas were sampled from 5.4 megapixel digital images. These areas were randomly sampled across all retinal layers in areas of detachment or similar control retina areas. For CD45 staining, the retina was selected as the region of interest from 20X field of view measuring 376,441 µm^2^. The total area of retinal CD45 immunofluorescence was calculated for regions with pixel intensities above a determined threshold and averaged per group. The average density was calculated as the mean pixel value above threshold within threshold-designated regions. The density sum was calculated as the total of pixel values for all pixels within threshold-designated regions. For TUNEL measurements, the number of immunofluorescent cells above threshold was determined for regions with pixel intensities above a determined threshold using Image-Pro 6.2. For outer nuclear layer thickness measurements, Image-Pro 6.2 was used to measure the height of the outer nuclear layer averaging evenly-spaced measurements taken per 20X field [71]. These calculations were determined for regions sampled from at least three different individuals for each experimental condition.

### Statistical Analysis

To determine whether differences in outer nuclear layer thickness, TUNEL-positive cells, and microglia in the detached retina at different timepoints were significant, analysis of variance (ANOVA) was first performed to confirm a difference in measurements with p<0.05 considered significant. Subsequent least squares means testing with Bonferroni correction was performed (p<0.007 was considered significant, with 7 conditions compared for ONL thickness and TUNEL, and p<0.0125 for microglia analysis with 4 conditions compared (RD to controls)). SAS v9.2 from the SAS Institute Inc. (Cary, NC) was used for analyses.

## References

[1] Agrawal RN, He S, Spee C, Cui JZ, Ryan SJ, et al (2007) In vivo models of proliferative vitreoretinopathy. Nat Protoc 2: 67–77. 17401340 10.1038/nprot.2007.4

[2] Mandava N, Blackburn P, Paul DB, Wilson MW, Read SB, et al (2002) Ribozyme to proliferating cell nuclear antigen to treat proliferative vitreoretinopathy. Invest Ophthalmol Vis Sci 43: 3338–3348. 12356843

[3] Schiff WM, Hwang JC, Ober MD, Olson JL, Dhrami-Gavazi E, et al (2007) Safety and efficacy assessment of chimeric ribozyme to proliferating cell nuclear antigen to prevent recurrence of proliferative vitreoretinopathy. Arch Ophthalmol 125: 1161–1167. 17846353 10.1001/archopht.125.9.1161

[4] Iribarne M, Ogawa L, Torbidoni V, Dodds CM, Dodds RA, et al (2008) Blockade of endothelinergic receptors prevents development of proliferative vitreoretinopathy in mice. Am J Pathol 172: 1030–1042. 18310504 10.2353/ajpath.2008.070605 PMC2276418

[5] Luna G, Kjellstrom S, Verardo MR, Lewis GP, Byun J, et al (2009) The effects of transient retinal detachment on cavity size and glial and neural remodeling in a mouse model of X-linked retinoschisis. Invest Ophthalmol Vis Sci 50: 3977–3984. 19387072 10.1167/iovs.08-2910 PMC2735860

[6] Fisher SK, Lewis GP, Linberg KA, Verardo MR (2005) Cellular remodeling in mammalian retina: results from studies of experimental retinal detachment. Prog Retin Eye Res 24: 395–431. 15708835 10.1016/j.preteyeres.2004.10.004

[7] Nakazawa T, Takeda M, Lewis GP, Cho KS, Jiao J, et al (2007) Attenuated glial reactions and photoreceptor degeneration after retinal detachment in mice deficient in glial fibrillary acidic protein and vimentin. Invest Ophthalmol Vis Sci 48: 2760–2768. 17525210 10.1167/iovs.06-1398 PMC2613948

[8] Lei H, Hovland P, Velez G, Haran A, Gilbertson D, et al (2007) A potential role for PDGF-C in experimental and clinical proliferative vitreoretinopathy. Invest Ophthalmol Vis Sci 48: 2335–2342. 17460299 10.1167/iovs.06-0965

[9] Hui YN, Goodnight R, Sorgente N, Ryan SJ (1989) Fibrovascular proliferation and retinal detachment after intravitreal injection of activated macrophages in the rabbit eye. Am J Ophthalmol 108: 176–184. 2757098 10.1016/0002-9394(89)90014-7

[10] Andrews A, Balciunaite E, Leong FL, Tallquist M, Soriano P, et al (1999) Platelet-derived growth factor plays a key role in proliferative vitreoretinopathy. Invest Ophthalmol Vis Sci 40: 2683–2689. 10509666

[11] Cook B, Lewis GP, Fisher SK, Adler R (1995) Apoptotic photoreceptor degeneration in experimental retinal detachment. Invest Ophthalmol Vis Sci 36: 990–996. 7730033

[12] Chang CJ, Lai WW, Edward DP, Tso MO (1995) Apoptotic photoreceptor cell death after traumatic retinal detachment in humans. Arch Ophthalmol 113: 880–886. 7605279 10.1001/archopht.1995.01100070054025

[13] Fisher SK, Lewis GP (2003) Muller cell and neuronal remodeling in retinal detachment and reattachment and their potential consequences for visual recovery: a review and reconsideration of recent data. Vision Res 43: 887–897. 12668058 10.1016/s0042-6989(02)00680-6

[14] Lewis GP, Fisher SK (2003) Up-regulation of glial fibrillary acidic protein in response to retinal injury: its potential role in glial remodeling and a comparison to vimentin expression. Int Rev Cytol 230: 263–290. 14692684 10.1016/s0074-7696(03)30005-1

[15] Lewis GP, Guerin CJ, Anderson DH, Matsumoto B, Fisher SK (1994) Rapid changes in the expression of glial cell proteins caused by experimental retinal detachment. Am J Ophthalmol 118: 368–376. 7916177 10.1016/s0002-9394(14)72962-9

[16] Sethi CS, Lewis GP, Fisher SK, Leitner WP, Mann DL, et al (2005) Glial remodeling and neural plasticity in human retinal detachment with proliferative vitreoretinopathy. Invest Ophthalmol Vis Sci 46: 329–342. 15623793 10.1167/iovs.03-0518

[17] Kroll AJ, Machemer R (1968) Experimental retinal detachment in the owl monkey. 3. Electron microscopy of retina and pigment epithelium. Am J Ophthalmol 66: 410–427. 4970987 10.1016/0002-9394(68)91524-9

[18] Anderson DH, Guerin CJ, Erickson PA, Stern WH, Fisher SK (1986) Morphological recovery in the reattached retina. Invest Ophthalmol Vis Sci 27: 168–183. 3943943

[19] Anderson DH, Stern WH, Fisher SK, Erickson PA, Borgula GA (1983) Retinal detachment in the cat: the pigment epithelial-photoreceptor interface. Invest Ophthalmol Vis Sci 24: 906–926. 6862795

[20] Erickson PA, Fisher SK, Anderson DH, Stern WH, Borgula GA (1983) Retinal detachment in the cat: the outer nuclear and outer plexiform layers. Invest Ophthalmol Vis Sci 24: 927–942. 6862796

[21] Lewis GP, Chapin EA, Luna G, Linberg KA, Fisher SK (2010) The fate of Muller’s glia following experimental retinal detachment: nuclear migration, cell division, and subretinal glial scar formation. Mol Vis 16: 1361–1372. 20664798 PMC2905639

[22] Nakazawa T, Hisatomi T, Nakazawa C, Noda K, Maruyama K, et al (2007) Monocyte chemoattractant protein 1 mediates retinal detachment-induced photoreceptor apoptosis. Proc Natl Acad Sci U S A 104: 2425–2430. 17284607 10.1073/pnas.0608167104 PMC1892947

[23] Lewis GP, Sethi CS, Carter KM, Charteris DG, Fisher SK (2005) Microglial cell activation following retinal detachment: a comparison between species. Mol Vis 11: 491–500. 16052164

[24] Hisatomi T, Sakamoto T, Sonoda KH, Tsutsumi C, Qiao H, et al (2003) Clearance of apoptotic photoreceptors: elimination of apoptotic debris into the subretinal space and macrophage-mediated phagocytosis via phosphatidylserine receptor and integrin alphavbeta3. Am J Pathol 162: 1869–1879. 12759244 10.1016/s0002-9440(10)64321-0 PMC1868143

[25] Kaneko H, Nishiguchi KM, Nakamura M, Kachi S, Terasaki H (2008) Characteristics of bone marrow-derived microglia in the normal and injured retina. Invest Ophthalmol Vis Sci 49: 4162–4168. 18487364 10.1167/iovs.08-1738

[26] Verardo MR, Lewis GP, Takeda M, Linberg KA, Byun J, et al (2008) Abnormal reactivity of muller cells after retinal detachment in mice deficient in GFAP and vimentin. Invest Ophthalmol Vis Sci 49: 3659–3665. 18469190 10.1167/iovs.07-1474 PMC2650509

[27] Lewis GP, Charteris DG, Sethi CS, Fisher SK (2002) Animal models of retinal detachment and reattachment: identifying cellular events that may affect visual recovery. Eye 16: 375–387. 12101444 10.1038/sj.eye.6700202

[28] Linberg KA, Sakai T, Lewis GP, Fisher SK (2002) Experimental retinal detachment in the cone-dominant ground squirrel retina: morphology and basic immunocytochemistry. Vis Neurosci 19: 603–619. 12507327 10.1017/s095252380219506x

[29] Jacobs GH, Calderone JB, Sakai T, Lewis GP, Fisher SK (2002) An animal model for studying cone function in retinal detachment. Doc Ophthalmol 104: 119–132. 11949805 10.1023/a:1014431701523

[30] Fischer AJ, Bongini R (2010) Turning Muller glia into neural progenitors in the retina. Mol Neurobiol 42: 199–209. 21088932 10.1007/s12035-010-8152-2

[31] Fischer AJ, Bongini R, Bastaki N, Sherwood P (2011) The maturation of photoreceptors in the avian retina is stimulated by thyroid hormone. Neuroscience 178: 250–260. 21256198 10.1016/j.neuroscience.2011.01.022 PMC3048918

[32] Fischer AJ, Omar G, Eubanks J, McGuire CR, Dierks BD, et al (2004) Different aspects of gliosis in retinal Muller glia can be induced by CNTF, insulin, and FGF2 in the absence of damage. Molecular vision 10: 973–986. 15623987

[33] Fischer AJ, Schmidt M, Omar G, Reh TA (2004) BMP4 and CNTF are neuroprotective and suppress damage-induced proliferation of Muller glia in the retina. Molecular and cellular neurosciences 27: 531–542. 15555930 10.1016/j.mcn.2004.08.007

[34] Fischer AJ, Scott MA, Zelinka C, Sherwood P (2010) A novel type of glial cell in the retina is stimulated by insulin-like growth factor 1 and may exacerbate damage to neurons and Muller glia. Glia 58: 633–649. 19941335 10.1002/glia.20950 PMC2830337

[35] Fischer AJ, Skorupa D, Schonberg DL, Walton NA (2006) Characterization of glucagon-expressing neurons in the chicken retina. J Comp Neurol 496: 479–494. 16572462 10.1002/cne.20937 PMC2565864

[36] Ghai K, Zelinka C, Fischer AJ (2010) Notch signaling influences neuroprotective and proliferative properties of mature Muller glia. J Neurosci 30: 3101–3112. 20181607 10.1523/JNEUROSCI.4919-09.2010 PMC2834965

[37] Mey J, Thanos S (2000) Development of the visual system of the chick. I. Cell differentiation and histogenesis. Brain Res Brain Res Rev 32: 343–379. 10760548 10.1016/s0165-0173(99)00022-3

[38] Thanos S, Mey J (2001) Development of the visual system of the chick. II. Mechanisms of axonal guidance. Brain Res Brain Res Rev 35: 205–245. 11423155 10.1016/s0165-0173(01)00049-2

[39] Kram YA, Mantey S, Corbo JC (2010) Avian cone photoreceptors tile the retina as five independent, self-organizing mosaics. PLoS One 5: e8992. 20126550 10.1371/journal.pone.0008992 PMC2813877

[40] Okano T, Kojima D, Fukada Y, Shichida Y, Yoshizawa T (1992) Primary structures of chicken cone visual pigments: vertebrate rhodopsins have evolved out of cone visual pigments. Proc Natl Acad Sci U S A 89: 5932–5936. 1385866 10.1073/pnas.89.13.5932 PMC402112

[41] Bruhn SL, Cepko CL (1996) Development of the pattern of photoreceptors in the chick retina. J Neurosci 16: 1430–1439. 8778294 10.1523/JNEUROSCI.16-04-01430.1996 PMC6578560

[42] Morris VB (1970) Symmetry in a receptor mosaic demonstrated in the chick from the frequencies, spacing and arrangement of the types of retinal receptor. J Comp Neurol 140: 359–398. 5476889 10.1002/cne.901400308

[43] Curcio CA, Sloan KR, Kalina RE, Hendrickson AE (1990) Human photoreceptor topography. J Comp Neurol 292: 497–523. 2324310 10.1002/cne.902920402

[44] Ochrietor JD, Moroz TP, Linser PJ (2010) The 2M6 antigen is a Muller cell-specific intracellular membrane-associated protein of the sarcolemmal-membrane-associated protein family and is also TopAP. Mol Vis 16: 961–969. 20577597 PMC2890575

[45] Fisher SK, Erickson PA, Lewis GP, Anderson DH (1991) Intraretinal proliferation induced by retinal detachment. Invest Ophthalmol Vis Sci 32: 1739–1748. 2032796

[46] Geller SF, Lewis GP, Anderson DH, Fisher SK (1995) Use of the MIB-1 antibody for detecting proliferating cells in the retina. Invest Ophthalmol Vis Sci 36: 737–744. 7890504

[47] Fischer AJ, Zelinka C, Scott MA (2010) Heterogeneity of glia in the retina and optic nerve of birds and mammals. PLoS One 5: e10774. 20567503 10.1371/journal.pone.0010774 PMC2887354

[48] Burt DW (2007) Emergence of the chicken as a model organism: implications for agriculture and biology. Poult Sci 86: 1460–1471. 17575197 10.1093/ps/86.7.1460

[49] Gerald H Jacobs, Jay Neitz, Crognale M (1985) Spectral sensitivity of ground squirrel cones measured with ERG flicker photometry. J Comp Physiol A 156: 503–509.

[50] Curcio CA, Allen KA (1990) Topography of ganglion cells in human retina. J Comp Neurol 300: 5–25. 2229487 10.1002/cne.903000103

[51] Kryger Z, Galli-Resta L, Jacobs GH, Reese BE (1998) The topography of rod and cone photoreceptors in the retina of the ground squirrel. Vis Neurosci 15: 685–691. 9682870 10.1017/s0952523898154081

[52] Headington K, Choi SS, Nickla D, Doble N (2011) Single cell imaging of the chick retina with adaptive optics. Curr Eye Res 36: 947–957. 21950701 10.3109/02713683.2011.587934 PMC5354302

[53] Bowmaker JK, Knowles A (1977) The visual pigments and oil droplets of the chicken retina. Vision Res 17: 755–764. 898682 10.1016/0042-6989(77)90117-1

[54] Lewis GP, Erickson PA, Anderson DH, Fisher SK (1991) Opsin distribution and protein incorporation in photoreceptors after experimental retinal detachment. Exp Eye Res 53: 629–640. 1835933 10.1016/0014-4835(91)90223-2

[55] Fariss RN, Molday RS, Fisher SK, Matsumoto B (1997) Evidence from normal and degenerating photoreceptors that two outer segment integral membrane proteins have separate transport pathways. J Comp Neurol 387: 148–156. 9331178 10.1002/(sici)1096-9861(19971013)387:1<148::aid-cne12>3.0.co;2-q

[56] Rex TS, Fariss RN, Lewis GP, Linberg KA, Sokal I, et al (2002) A survey of molecular expression by photoreceptors after experimental retinal detachment. Invest Ophthalmol Vis Sci 43: 1234–1247. 11923271

[57] Rex TS, Lewis GP, Geller SF, Fisher SK (2002) Differential expression of cone opsin mRNA levels following experimental retinal detachment and reattachment. Mol Vis 8: 114–118. 11979236

[58] Nork TM, Millecchia LL, Strickland BD, Linberg JV, Chao GM (1995) Selective loss of blue cones and rods in human retinal detachment. Arch Ophthalmol 113: 1066–1073. 7639660 10.1001/archopht.1995.01100080118039

[59] Sakai T, Calderone JB, Lewis GP, Linberg KA, Fisher SK, et al (2003) Cone photoreceptor recovery after experimental detachment and reattachment: an immunocytochemical, morphological, and electrophysiological study. Invest Ophthalmol Vis Sci 44: 416–425. 12506104 10.1167/iovs.02-0633

[60] Davidorf FH, Havener WH, Lang JR (1975) Macular vision following retinal detachment surgery. Ophthalmic Surg 6: 74–81. 1208038

[61] Ross WH, Kozy DW (1998) Visual recovery in macula-off rhegmatogenous retinal detachments. Ophthalmology 105: 2149–2153. 9818620 10.1016/S0161-6420(98)91142-3

[62] Ross WH (2002) Visual recovery after macula-off retinal detachment. Eye (Lond) 16: 440–446. 12101451 10.1038/sj.eye.6700192

[63] Diederen RM, La Heij EC, Kessels AG, Goezinne F, Liem AT, et al (2007) Scleral buckling surgery after macula-off retinal detachment: worse visual outcome after more than 6 days. Ophthalmology 114: 705–709. 17194479 10.1016/j.ophtha.2006.09.004

[64] Hassan TS, Sarrafizadeh R, Ruby AJ, Garretson BR, Kuczynski B, et al (2002) The effect of duration of macular detachment on results after the scleral buckle repair of primary, macula-off retinal detachments. Ophthalmology 109: 146–152. 11772595 10.1016/s0161-6420(01)00886-7

[65] Salicone A, Smiddy WE, Venkatraman A, Feuer W (2006) Visual recovery after scleral buckling procedure for retinal detachment. Ophthalmology 113: 1734–1742. 17011955 10.1016/j.ophtha.2006.03.064

[66] Gharbiya M, Grandinetti F, Scavella V, Cecere M, Esposito M, et al (2012) Correlation between spectral-domain optical coherence tomography findings and visual outcome after primary rhegmatogenous retinal detachment repair. Retina 32: 43–53. 21778929 10.1097/IAE.0b013e3182180114

[67] Cuadros MA, Santos AM, Martin-Oliva D, Calvente R, Tassi M, et al (2006) Specific immunolabeling of brain macrophages and microglial cells in the developing and mature chick central nervous system. The journal of histochemistry and cytochemistry : official journal of the Histochemistry Society 54: 727–738. 16461367 10.1369/jhc.5A6832.2006

[68] Wenningmann I, Dilger JP (2001) The kinetics of inhibition of nicotinic acetylcholine receptors by (+)-tubocurarine and pancuronium. Mol Pharmacol 60: 790–796. 11562442

[69] Pilar G, Nunez R, McLennan IS, Meriney SD (1987) Muscarinic and nicotinic synaptic activation of the developing chicken iris. J Neurosci 7: 3813–3826. 2826718 10.1523/JNEUROSCI.07-12-03813.1987 PMC6569112

[70] Ghai K, Stanke JJ, Fischer AJ (2008) Patterning of the circumferential marginal zone of progenitors in the chicken retina. Brain Res 1192: 76–89. 17320838 10.1016/j.brainres.2007.01.105 PMC2775427

[71] Cebulla CM, Ruggeri M, Murray TG, Feuer WJ, Hernandez E (2010) Spectral domain optical coherence tomography in a murine retinal detachment model. Exp Eye Res 90: 521–527. 20114045 10.1016/j.exer.2010.01.008 PMC3701460

[72] Jonas JB, Schneider U, Naumann GO (1992) Count and density of human retinal photoreceptors. Graefes Arch Clin Exp Ophthalmol 230: 505–510. 1427131 10.1007/BF00181769

